# Mr. Urvins, on Venæsection, &c.

**Published:** 1800-03

**Authors:** David Urvins

**Affiliations:** Grenville-Street, London


### *To the Editors of the Medical and Physical Journal.*

GENTLEMEN,

IF upon a perusal of the inclosed, you find it worthy a place in your valuable publication, the author will find himself extremely gratified.

He is, very respectfully,

Your obedient servant,

DAVID URVINS.

*Grenville-Street, London,*

*Jan. 20, 1800.*

---

The progress of every science, and particularly that of Medicine, has been, in all ages, retarded by the *enthusiasm* of its professors.

Calm reasoning, cool deliberation, unprejudiced opinions, are qualities equally rare and desirable.

It peculiarly behoves the professors of the most important of all sciences, to guard their minds against the reception of hasty hasty and extravagant notions, either with regard to the theory or practice of medicine.

The general prevalence of infidelity and scepticism has been, with some degree of justice, attributed to enthusiasm in religion; and, perhaps, *Medical Enthusiasm*, (if the term is allowed,) has been attended with more evils than mankind in general are aware of.

Let it not be supposed, that in recommending moderation, the writer of this paper is an advocate for *indifference*, *inactivity*, or *negligence*; nothing is more remote from his design, than a wish to check either ardour or perseverance; but the axiom of Horace, "*Est modus in rebus*," should never, for a moment, be forgotten.

These observations have been elicited by the perusal of a paper which appeared in the *Medical and Physical Journal*, for *January*, written by Mr. *Huggan*.

It would be the extreme of injustice, to deny this gentleman the merit of ingenuity and piquancy in his communications; and I cannot but acknowledge myself very considerably indebted to him for some of his observations.

No one, however, who gives the subject the smallest consideration, will deny the extreme impropriety of advancing the following assertion. Mr. *Huggan* says, when treating of Venæsection, "that this practice is *never necessary*, seldom safe, "often hurtful, and sometimes fatal."

Perhaps the *comparative disuse* of the *lancet*, is one of the greatest improvements in the *Modern Practice of Physic*. With great justness and veracity may it be said, that *fevers* have been fatal to thousands, and *Venæsection*, in *these disorders*, to tens of thousands.

The *Sangrado Practice* of universal and indiscriminate depletion and relaxation, is now happily exploded by discerning and scientific practitioners; but the most useful article in the whole *Materia Medica*, might, with equal justice, be pronounced destitute of utility, as the practice of blood-letting.

The injudicious administration of *opium*, by which many valuable lives have been shortened, will not deter the medical practitioner from continuing to exhibit this useful medicine; those, however, who have had occasion to witness the fatal effects of its misapplication, would be equally justified with Mr. *H.* in recommending its complete expulsion from the list of remedies.

May I be permitted to add a few words in favour of the practice, which this gentleman has recommended in *Synocha*?

The general inflammatory symptoms which have character-

 ized ized this disease, have induced Physicians to omit the use of *opium*. This omission has arisen in consequence of mistaking the mere *effect* of the disease for the *disease* itself.

Mr. Huggan has very properly remarked, that the seat of the disorder, which has obtained the name (though, perhaps, erroneously) of Inflammatory Fever, is in the *Sentient System*; and as the operation of opium is now pretty generally acknowledged to be confined to the *Nerves*, its exhibition cannot fail of being attended with success in Synocha.

In support of this theory, the writer of the present article is very happy in having it in his power to adduce his own experience; as his practice has afforded him frequent opportunities of witnessing the beneficial effects of this valuable medicine in Inflammatory, and in *every other species of Fever*.

---

